# Polynomial Multiple Regression Analysis of the Lubrication Effectiveness of Deep Drawing Quality Steel Sheets by Eco-Friendly Vegetable Oils

**DOI:** 10.3390/ma15031151

**Published:** 2022-02-02

**Authors:** Tomasz Trzepieciński

**Affiliations:** Department of Manufacturing and Production Engineering, Faculty of Mechanical Engineering and Aeronautics, Rzeszow University of Technology, al. Powst. Warszawy 8, 35-959 Rzeszow, Poland; tomtrz@prz.edu.pl

**Keywords:** deep drawing, metal forming, sheet metal forming, steel sheets, sustainable manufacturing, vegetable oils

## Abstract

Ensuring adequate lubrication is a key task in the sheet metal forming process. The replacement of commonly used synthetic lubricants in metal forming operations by eco-friendly equivalents is a way to introduce sustainable manufacturing. In this paper, six kinds of vegetable oils (linseed, palm, sunflower, cotton seed, soybean and coconut) were used to study the effect of lubricant type on the value of the coefficient of friction (COF) in sheet metal forming. The strip drawing test was used to simulate the friction conditions. The tests were carried out for various lubrication conditions and pressures. The polynomial quadratic regression model was used to determine the relationship between the input variables (test conditions) and the COF. For the range of the nominal pressures considered (2–12 MPa), the following oils provided the highest lubrication efficiency: palm, sunflower and cotton seed. These oils decreased the value of the COF by about 11–16% depending on the nominal pressure. Linseed oil had the most unfavourable properties, reducing the COF by about 7–12%. For the whole range of pressures considered, the increase in the viscosity of the oil caused a reduction in the value of the COF. The effect of oil density on the COF value was similar. The most unfavourable friction conditions occurred when there was low density and low viscosity of the oil at the same time.

## 1. Introduction

Sheet metal forming (SMF) is a common method of obtaining products with a complex shape, primarily in the automotive industry [1,2]. During this process, the sheet is deformed with metallic dies of a shape corresponding to the shape of the product. During stamping, the sheet is deformed by exceeding the yield point of its material. The increase in the strength of the drawpiece is related to the work hardening of the sheet material [3].

The main factors influencing the tribological phenomena in SMF processes are, among others, the kinematics of the tool movement, the character of the loads (static and dynamic contact), the micro- and macro-geometry of the contact, the physicochemical phenomena on the contact surface and the temperature [4,5]. Friction in SMF processes is a complex function of the process parameters, the surface topography of the sheet to be formed and the tools, and the contact conditions that are constantly evolving in various regions of the drawpiece [6]. One of the methods for effectively reducing the friction during SMF is lubricating the surface of the sheet formed with greases [7,8].

In order to improve some operational properties of plastic greases, improvers are added to them in the production process. Improvements are chemical compounds, organic or inorganic, causing the appearance or improvement of the desired functional properties of the lubricant. The main task of these additives is to reduce the friction and wear of the lubricated surfaces and to prevent decomposition of the lubricant and corrosion of the component surface, and deposition of the wear products and lubricant decomposition [9]. A large number of SMF processes and a wide variety of their implementation parameters make the range of technological lubricants used very large [10,11]. Due to the method for applying the lubricant to the surface, there are solid and liquid lubricants, as well as spray ones. In addition to petroleum-based synthetic lubricants, vegetable lubricants have been very popular in recent years [12,13]. Natural-origin lubricants have good lubricating properties, mainly due to their specific chemical structure [14,15]. Vegetable oils have long hydrocarbon chains and functional groups, such as carboxyl or amine, and often also a double bond. Long fatty acids enable the effective separation of the tool/workpiece interfaces under boundary friction regimes [16]. Vegetable-based lubricants have exclusive properties, such as a high viscosity index and flash point, high lubricity, low evaporation rate and polar nature [17,18]. One of the most important physicochemical properties of lubricants is their wettability, which determines the intensity of lubricant absorption on the surface of the material formed or on a tool [19,20]. Vegetable oils offer 95% biodegradability, which helps in reducing the cost of disposal [21]. Vegetable bio-lubricants are eco-friendly due to their non-toxicity, net zero greenhouse gas emission and biodegradability [22].

Experimental tests simulating friction and lubrication conditions can be divided into tests simulating processes and tests simulating tribological conditions [23]. Many tribological tests have been developed to determine the coefficient of friction (COF) and/or their evolution during the forming process. These tests include the strip drawing test (SDT) [24,25], the bending under tension (BUT) test [26] and the draw bead test (DBT) [27]. To determine the COF value, tribotesters are also used, which are typically intended for wear testing, that is, pin-on-disc and ball-on-disc tests. Recent developments in friction testing for conventional SMF and incremental sheet forming are presented in the review paper by Trzepieciński and Lemu [23].

In recent years, the SDT, as the simplest and quickest method of determining the COF, has been of interest to many authors [25,28,29]. Trzepieciński and Fejkiel [24] investigated the effect of sheet deformation on changes to the surface roughness parameters and the friction coefficient value of deep drawing quality steel sheets in the SDT. It was found that the COF of pre-strained samples decreases with increasing nominal pressure for both lubricated and dry conditions. Trzepieciński [25] compared the results of the COF of DC04 steel sheets in three commonly used friction tests, that is, SDT, DBS and BUT. Jewvattanarak et al. [30] investigated the tribological performance of four different lubrication conditions (dry friction, chlorine additive lubricant, chlorine-free lubricant and mineral base oil) in the SDT. They found that the combination of chlorine and sulphur additives provided the best tribological behaviours. Filzek et al. [31] conducted an analysis of temperature-induced friction increase in an SDT. They considered different tribological systems at tool temperatures between 20 and 80 °C. It was found that the temperature increase results in a higher friction of up to 77%. Prakash and Kumar [32] analysed the performance of three vegetable lubricants (Jatropha, Karanja and cotton seed oils) by applying the SDT to test an AA5083-O alloy sheet. SDTs were conducted to determine the COF. Cotton seed oil was found to be better than the other two oils. The bio-lubricants tested were found to perform well in comparison to mineral-oil-based lubricants in terms of the resulting friction coefficient. Le and Sutcliffe [33] used the SDT to investigate the friction behaviour under thin film lubrication in SMF. Surface topography analysis of 1050 aluminium alloy sheets showed that the change in the COF is associated with a change in the contact ratio between the strip and the tool. Evin and Tomáš [34] tested high-strength TRIP and extra deep drawing quality DX54D steel sheets in an SDT to determine the COF under lubrication when using thixotropic lubricating oil. They concluded that the COFs are not constant and depend on the pressure on the die contact surfaces.

Currently, over 10,000 different mineral and synthetic lubricants are used [35]. However, the significant health risks and increased costs of mineral oils have caused a global shift in promoting green alternatives as potential substitutes. Environmentally friendly, non-toxic and biodegradable vegetable oils [36] with better tribological properties than conventional mineral oil [37,38] are considered as a substitute for petroleum-based lubricants [39]. As the world is facing food crises, the source of vegetable oils can be seen in withdrawn production batches or withdrawn oils as the expiry date has been exceeded. These oils are not suitable for human consumption, but may be a source of lubricant in metal forming before recycling.

Research into the use of vegetable lubricants in SMF operations is not yet widespread in the scientific community. One of the reasons for this is the use of lubricants and procedures developed over many years, especially in the large-series industry. Approximately 85% of lubricants being used around the world are petroleum-based oils [40,41]. Changing a lubricant in terms of implementing sustainable manufacturing usually involves a change in technology and is costly. Nevertheless, as shown in the literature review, biodegradable vegetable oils are of interest to scientists. The lubrication efficiency of vegetable lubricants in the processing of the deep drawing steel sheets commonly used in the automotive industry has not been sufficiently researched so far and requires further work. In this paper, the lubrication performance of six typical vegetable lubricants with a wide range of viscosities was studied. A specially designed tribotester, which simulates the SDT, a very popular method used in the forming technologies to determine the COF [25,28,29], was used for this purpose. Due to the complex interactions between the lubricant properties and nominal pressures, and the COF as an output variable, polynomial quadratic regression analysis was used to analyse the results.

## 2. Material and Methods

### 2.1. Material

Deep drawing quality cold rolled steel sheets with 0.8 mm thickness were used as the test material. The chemical composition of the DC04 steel sheet (≤0.4% Mn, ≤0.08% C, ≤0.03% *p* ≤ 0.03% S, Fe—remainder) is required by the EN 10130 [42] standard. Due to its high formability and strength, a DC04 steel sheet is used where rigidity, ductility and strength are required [43]. Typical applications are found in the domestic appliance sector, radiators, small welded tubes, metal furniture, drums and the automotive industry.

**Figure 1:**
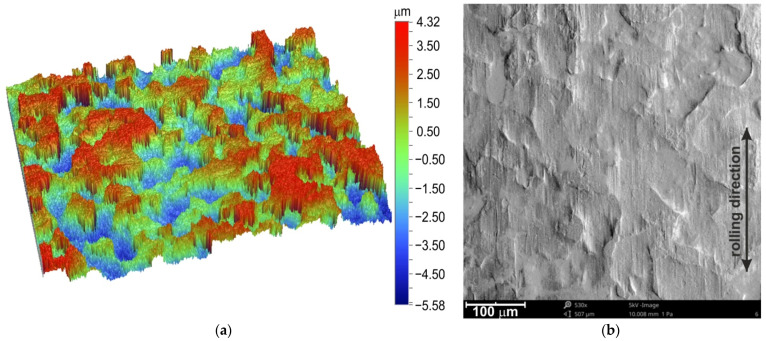
(**a**) Topography and (**b**) SEM micrograph of the original surface of the DC04 steel sheet.

The surface roughness parameters of the tested sheet were as follows: average roughness Sa = 1.31 μm; root mean square roughness Sq = 1.53 μm; kurtosis Sku = 2.10; skeweness Ssk = −0.13; the maximum height Sz = 20.79 μm; maximum pit depth Sv = 10.31; and root mean square gradient Sdq = 0.14. A Talysurf CCI Lite 3D optical profiler was used to characterise the surface roughness of the sheets. The measurement of the surface topography (Figure 1a) was carried out using a Bruker Contour GT 3D optical microscope.

The original surface and surface topographies were examined using an S-3400 scanning electron microscope (SEM) from PhenomProX. The SEM micrograph of the original surface (Figure 1b) revealed the directional topography resulting from the rolling process.

### 2.2. Strip Drawing Test

**Figure 2:**
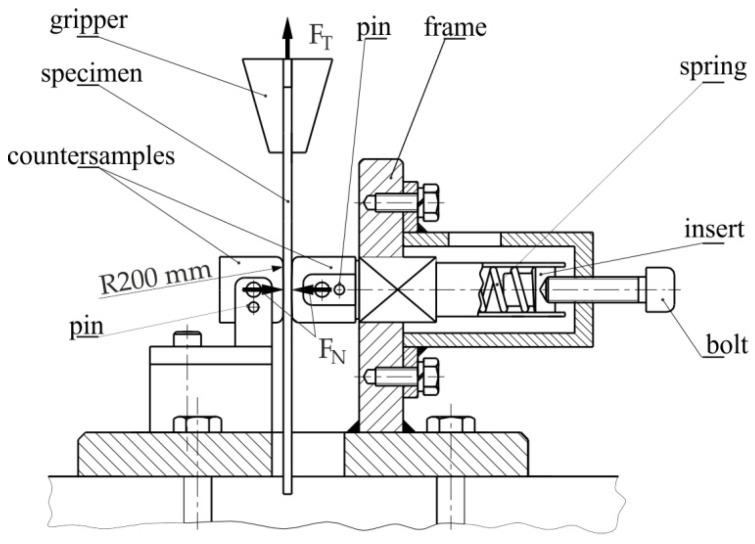
Cross-section of the device.

Figure 2 shows the cross-section of the tribological simulator of the SDT. The test consisted of pulling long strips (20 mm wide and 400 mm long) of sheet metal through a system of two countersamples made of cold-work tool steel, corresponding to the steel used for real stamping dies. All samples were cut along the rolling direction. The rounding radius of the countersamples was r = 200 mm (Figure 2). The basic surface roughness parameters of the countersamples were as follows: average roughness Sa = 1.53 μm; skeweness Ssk = −0.014; and kurtosis Sku = 2.07. The upper end of the sample was mounted in the upper gripper of a Zwick/Roell Z100 universal tensile testing machine. In turn, the frame of the device was mounted in the lower holder of the universal testing machine.

The aim of this research was to determine the friction conditions prevailing in the industrial practice of SMF. According to Cillauren et al. [44], who conducted a wide literature review, the range of contact pressures covered by different authors is within 1–15 MPa. Due to the constructional limitations of the device in reproducing such a range of pressures, the range of pressures examined in this research was limited to 1–12 MPa. Taking into account the geometry of the countersamples, such contact pressures require relatively small pressure forces. Therefore, the normal force F_N_ was exerted with a spring of the correct stiffness and set bolt. The normal force exerted on the specimen was obtained by suitable deflection of the spring. The relationship between the displacement and the force of the spring deflection in the range of 1 and 16 mm was determined using a MultiTest 10-i testing machine. The approximation of the force–displacement characteristics resulted in R^2^ = 0.990. The deflection of the spring was realised by rotating the bolt head considering that the pitch of the thread was equal to 1.25 mm.

**Figure 3:**
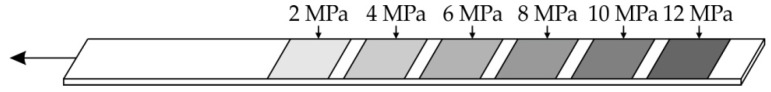
Schematic representation of the levels of nominal pressure.

After placing the specimen between the countersamples and fixing the upper end of the specimen in the holder of the testing machine, the lowest considered contact pressure was set. After activating the movement of the upper holder of the testing machine, the samples were pulled over a distance of about 20 mm. Then, the nominal pressure was increased to the next level, and the sample was pulled again over the same distance. The operations were repeated for all considered nominal pressures of 2, 4, 6, 8, 10 and 12 MPa. Therefore, one sample permitted the determination of the values of the friction coefficient for six levels of nominal pressure (Figure 3). For the tests carried out at all levels of nominal pressure, the sheet was drawn for a distance of about 20 mm. Seven strip samples were used to investigate the COF of the DC04 sheet at various friction conditions (six oils and dry friction conditions). The value of the tensile force F_T_ was recorded through the load cell of the measuring test machine and registered by testXpert^®^ testing software. Friction tests were carried out at a constant speed of 10 mm/s [24] at a temperature of 25 °C.

The value of the COF μ was determined as the ratio of the friction force to the pressure force:

(1) $$\mu =\frac{F_{T}}{2F_{N}}$$

where F_N_ is the normal (pressure) force and F_T_ is the friction force.

The coefficient of friction was evaluated separately for all levels of variation of pressure force F_N_. For each of these ranges, we obtained about 250–350 discrete values of the COF. The mean COF was determined using the formula [24]:

(2) $$\mu_{m}=\frac{1}{i}\underset{i}{\sum }\mu_{i}$$

where i is the number of friction coefficients determined for the specific level of constant normal force.

The nominal pressure p was determined based on the normal force of the countersamples using the relationship [24]:

(3) $$p=\sqrt{\frac{0.418^{2}{\;F}_{N}\cdot E}{b\cdot R}}$$

where R is the radius of the countersample surface, F_N_ is the normal force of the countersamples, b is the width of specimen and E = 205,000 MPa is the Young’s modulus of the sheet material.

The lubrication performance of six typical vegetable lubricants with a wide range of kinematic viscosity was studied. The analysis of variance for a wide range of changes in kinematic viscosity would allow us to better identify the influence of the process parameters on the value of the friction coefficient. The kinematic viscosity η_k_ of the most commonly used vegetable lubricants varies between 27 and 40 mm^2^/s. Therefore, the most common oils with a kinematic viscosity in this range were selected for testing. The following vegetable oils were selected: palm, sunflower, cotton seed, soybean, linseed and coconut. Moreover, to check the effectiveness of the lubrication, a reference test in dry friction conditions was conducted.

Prior to testing, both sides of the specimen were degreased using acetone, and after drying, they were oiled by a roller system that allows a uniform oil coating between 1.5 and 2 g·m^−2^ to be obtained, which was comparable to the stamping process conditions [45].

### 2.3. Analysis of Variance

The analysis of variance (ANOVA) is the analysis of the relationship between the variables in order to build a model that reflects this relationship well. In this article, a polynomial quadratic regression model was used to analyse the effect of the process parameters on the value of the COF. In the ANOVA, the following factors were included: kinematic viscosity, oil density and nominal load (Table 1). Vegetable oils had a similar density. However, oils showing a similar or the same density were characterized by different viscosity values. Therefore, the author’s intention was to check the synergistic effect of both these parameters simultaneously. Determining such a dependence without analysis of variance would be practically impossible. Moreover, nominal load is a parameter that directly affects the value of the friction coefficient, even more so than the lubrication conditions [15,19,24,25]. The explained variable was the value of the COF.

The selection of explained variables is, apart from the choice of regression method, a key task affecting both the fit and accuracy of the regression model forecasts. It is also very important to identify whether the dependence of the selected predictors on the explained variable is linear. With large non-linearities, the quality of the models obtained, despite the correct selection of explanatory variables, may be unsatisfactory [46]. In such cases, in the regression equation it is possible to introduce additional predictors obtained from the original variables with the use of appropriately selected nonlinear functions.

The backward elimination method used in this paper to check the significance of variables was based on the F statistic. The procedure starts with fitting a polynomial with a high degree, for example, p. Then, the H_0_ hypothesis is tested using the F statistic to see if the coefficient a_p_ (the degree of the polynomial) at the highest power is zero. The H_0_ hypothesis states that the observed sequence of observations x_1_, …, x_n_ comes from the sample with density f_0_(x). If the test result is positive, the highest coefficient is eliminated by lowering the degree of the polynomial by one. The procedure is repeated successively for decreasing degrees of polynomials, until a negative answer is obtained [47].

At each backward elimination step, the independent variable with the highest probability corresponding to the Fisher parameter F is removed from the model if the probability is high enough. In the ANOVA analysis, variables were removed if the probability was not less than *p* = 0.100. The test of the significance of the regression model was performed by calculating the ratio of the mean regression square and the mean square error (F statistics) at the significance level α = 0.05.

## 3. Results and Discussion

### 3.1. Effectiveness of Lubrication

In sheet metal forming, the lubrication efficiency depends on the lubricating properties of the oil, the range of the values of the forces acting on the lubricated friction couple and the properties of the surface layer, especially the roughness of the surfaces in contact with each other. These decide the relationship between the conditions in the surface micro-areas and the forces acting on the friction couple. The surface roughness is, therefore, the cause of the differences in pressures occurring in the surface asperities. To determine the effectiveness of the lubricants used to reduce the frictional resistance, let us introduce the concept of the lubrication efficiency coefficient λ_l_:

(4) $$\lambda_{l}=\frac{\mu_{d}-\mu_{l}}{\mu_{d}}\cdot 100\%$$

where μ_d_ is the coefficient of friction determined in dry friction conditions and μ_l_ is the COF determined in lubricated conditions.

**Figure 4:**
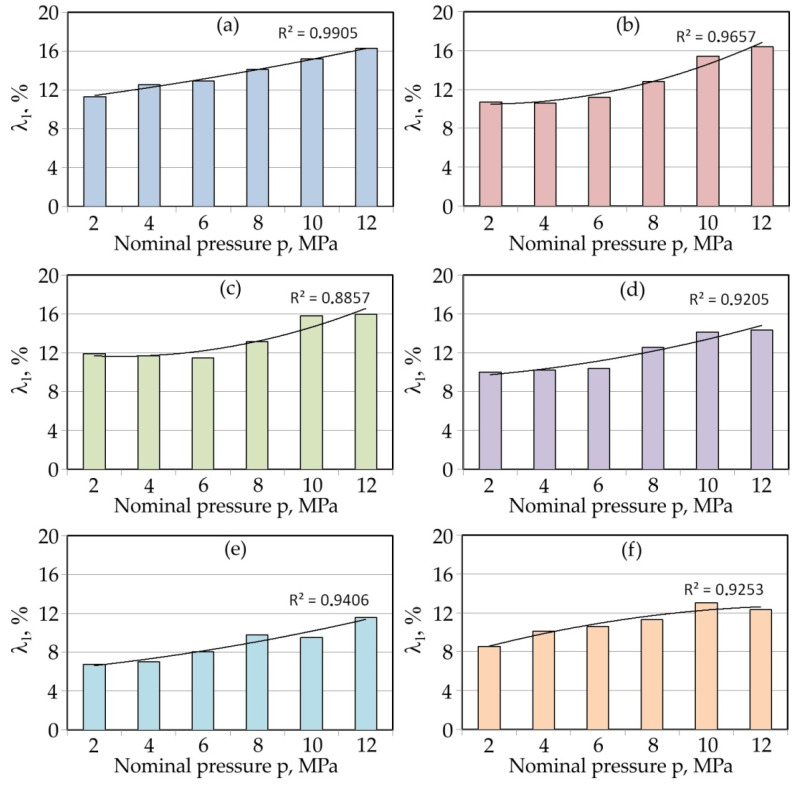
Effectiveness of the lubrication of (**a**) palm oil, (**b**) sunflower, (**c**) cotton seed oil, (**d**) soybean oil, (**e**) linseed oil and (**f**) coconut oil.

Figure 4 shows the lubrication efficiency for all the analysed lubricants. This figure also shows the trend lines for the changes in the λ_l_-value. These are second degree polynomials. Palm oil (Figure 4a), sunflower oil (Figure 4b) and cotton seed oil (Figure 4c) showed a similar value of lubrication efficiency in a range between 11 and 16% according to the nominal pressure. Soybean oil (Figure 4d) showed slightly less favourable lubricating properties. On the other hand, linseed oil (Figure 4e) and coconut oil (Figure 4f) had the lowest ability to reduce the value of the COF. Their lubrication efficiencies did not exceed 7% and were about 12% for the lowest and highest nominal pressure, respectively. In general, the lubrication efficiency increased with increasing nominal pressure.

The load of the countersample with a much higher hardness than the material of the deformed plate caused the flattening of the peaks of the asperities. During contact, the surface roughness of the cooperating elements changed until the so-called equilibrium roughness was reached. As was found by Trzepieciński et al. [48], during the loading of the rough sheet material, only the asperities of the surface were deformed to some extent. Increasing the normal load caused plastic deformation occurring in the subsurface, some distance below the roughness profile, while the surface asperities did not undergo further deformation. This is related to the high resistance to displacement of the sheet metal resulting from the interpenetration of the tool and sheet metal surface asperities.

**Figure 5:**
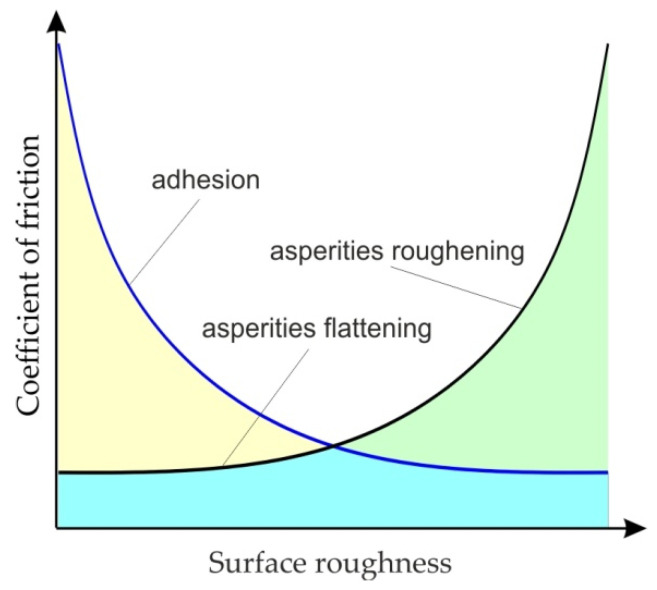
Effect of the surface roughness of the sheet metal on the COF.

The frictional resistances which exist in SMF under lubricated conditions depend, however, on the interaction between two mechanisms: adhesion and flattening/roughening of the asperities. The occurrence of these two mechanisms and their intensity depends on the surface roughness of the sheet (Figure 5). With low roughness of the sheet, the dominant mechanism is the adhesion of surfaces, while during the friction of high roughness surfaces, the mechanism of the asperities roughening dominates, and the intensity of this mechanism increases with increasing roughness of the surface of the deformed sheet with a much lower hardness than the tool material.

**Figure 6:**
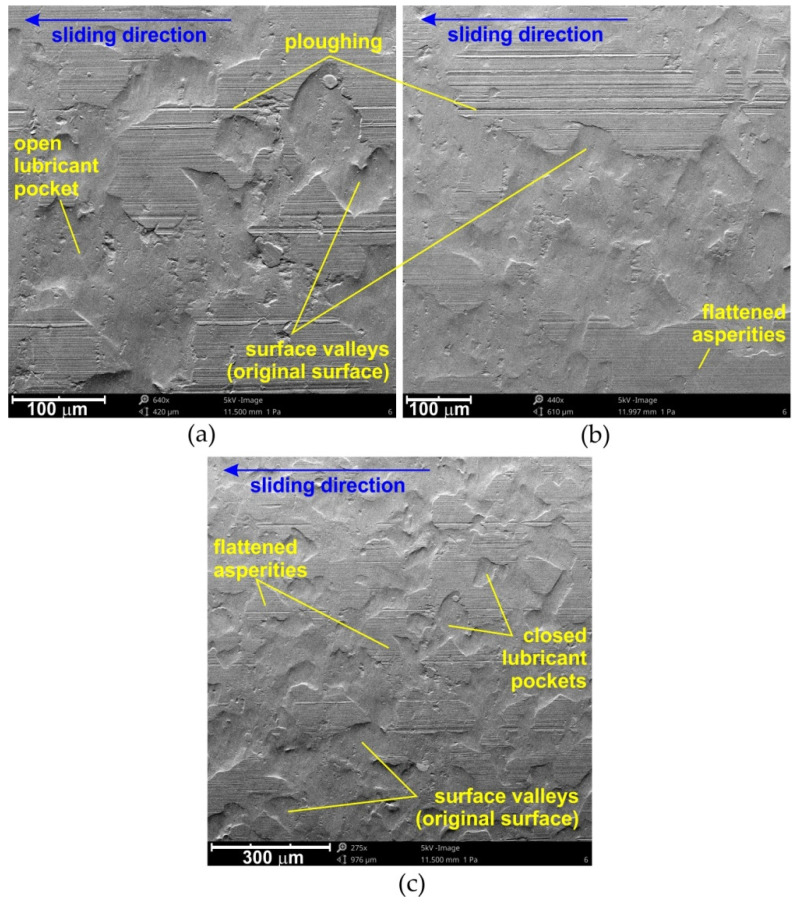
SEM micrographs of the specimen surfaces tested at 10 MPa in lubricated conditions using (**a**) cotton seed oil, (**b**) linseed oil and (**c**) coconut oil.

Figure 6 shows the SEM micrographs of the specimen surfaces tested at 10 MPa in lubricated conditions for cotton seed, linseed and coconut oils. For the remaining oils tested, the features in the surfaces subjected to friction were similar. The analysis of the SEM micrographs of the sheets showed the formation of closed lubricant pockets (Figure 6c), which, as lubricant reservoirs, created a lubricant cushion that reduced the COF value.

In such conditions, the intensity of lowering the frictional resistance increased with the increase in pressure, which is in line with the results presented in Figure 4. Roughness valleys around the edge of the surface are called open lubricant pockets as they cannot hold the lubricant [49,50]. The increased friction in microforming was mainly attributed to the increasing ratio of open lubricant pockets with the decreasing specimen size [49]. A mixed lubrication regime is common in typical sheet forming operations and is governed by the direct asperity contact, as well as the hydrodynamic pressure in the closed oil pockets developed by the lubricant [8,51].

**Figure 7:**
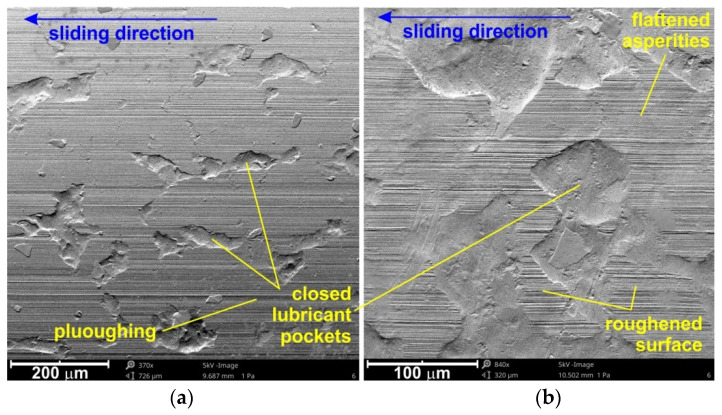
SEM micrographs of the specimen surfaces tested at 12 MPa in dry friction at different magnifications: (**a**) ×370 and (**b**) ×840.

Along with increasing contact pressure, favourable conditions may occur for the formation of elastohydrodynamic lubrication, taking into account the elastic deformation of the surface asperities, as well as a change in the lubricant viscosity caused by the pressure increase in the micro-contact areas [52,53]. An indispensable process accompanying friction is surface wear, which takes place in the area of roughness asperities (Figure 6a–c). While at relatively low nominal pressures, flattening was the the dominant mechanism, ploughing (Figure 7a) and roughening (Figure 7b) mechanisms emerged with increasing pressure, especially in dry friction conditions.

### 3.2. Analysis of Variance

Table 2 shows the results of the ANOVA for the coefficient of friction. The model F-value of 78.41 implied that the model was significant. Statistically insignificant factors that affected the process were above the *p*-value of 0.100. As the value of the *p*-value for density slightly exceeded the limit value, density was an insignificant factor. However, density correlated with viscosity (AB) was a statistically significant parameter. A *p*-value below 0.05 indicated that the viscosity and pressure were significant factors in the friction process. Viscosity is a key parameter that affects the coefficient of friction.

The final equation in terms of the coded factors is as follows (by default, the low levels are coded as −1 and the high levels of the factors are coded as +1):

COF = 0.2159 − 0.0051A − 0.0093B − 0.0063C − 0.0095AB + 0.0027B^2^ − 0.0015C^2^ (5)

The function that describes the COF is given in Equation (6) in terms of actual factors:

COF = −3.17144 + 3.80039A + 0.111843B − 0.000413C − 0.127361AB + 0.000069B^2^ − 0.00006C^2^ (6)

The total correlation R^2^ of the regression model was equal to 0.9419 (Table 3). Due to the small difference between the predicted R^2^ (0.9112) and the adjusted R^2^ (0.9299), it can be concluded that the model is adequate. The signal-to-noise ratio parameter value for a realistic model should be greater than 4. The calculated value of this coefficient in the model was over 33.9, so the regression model is adequate.

**Figure 8:**
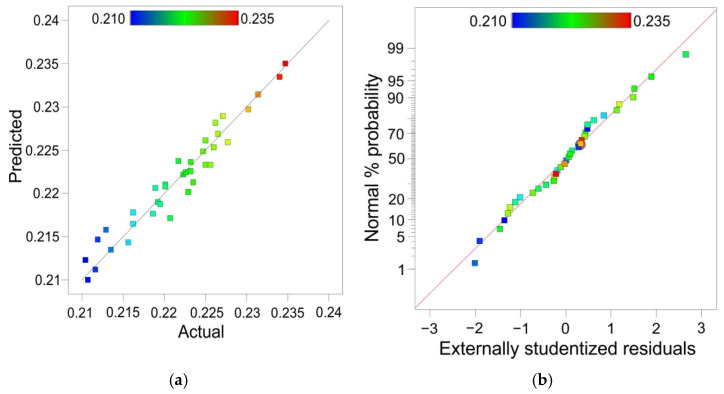
(**a**) The predicted vs. actual values of coefficient of friction and (**b**) the normal probability plot of the externally studentised residuals.

**Figure 9:**
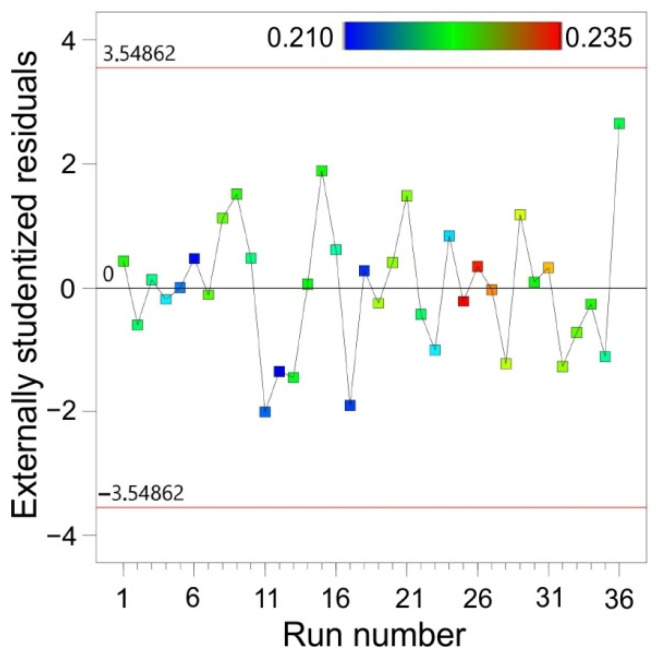
Distribution of the externally studentised residuals through the run number.

A comparison of the experimental values of the maximum formable wall angle with the values predicted by the ANOVA model is presented in Figure 8a. The distribution of externally studentised residuals along the line (Figure 8b) showed that the distribution of residuals in the model was normal. The normal distribution of the residuals was necessary to verify the significance of the parameters obtained. Externally studentised residuals for all experiments were distributed proportionally to the zero line (Figure 9).

Outliers may reflect the actual distribution or could be the result of an incident, but may also be the result of a measurement error. A large number of outliers can also be a signal that the wrong model has been chosen. Cook’s distance is a measure of the degree of change in the regression coefficients, if the given case was omitted from the calculations. Cook’s distances are a combined measure of the effect of individual observations on the regression line; that is, they measure the distance of the standard variables from the regression line and are a measure of the distance of the cases from the centre of gravity determined by the independent variables. All values of Cook’s distances should be of the same order. Otherwise, the given case probably has a significant influence on the weighting of the regression equation.

Cook’s distance measures the change in the values of the regression coefficients when the i-th observation is omitted:

(7) $$D_{i}=\frac{h_{i}}{{\left(1-h_{i}\right)}^{2}}\cdot \frac{e_{i}^{2}}{k\cdot \mathrm{MSE}}$$

where the first fraction is the influence measure and the second fraction is the variability measure, e_i_ is residual, k is number of parameters in the regression equation and MSE is the mean square error of the model.

The value of h_i_ measures the distance of a given observation from the mean value of the variable x:

(8) $$h_{i}=\frac{1}{N}+\frac{{\left(x_{i}-\;\bar{x}\right)}^{2}}{\sum_{i=N}^{N}{\left(x_{i}-\bar{x}\right)}^{2}}$$

where N is the number of observations, and $x_{i}-\bar{x}$ is a measure of deviation observation x_i_ from $\bar{x}$.

**Figure 10:**
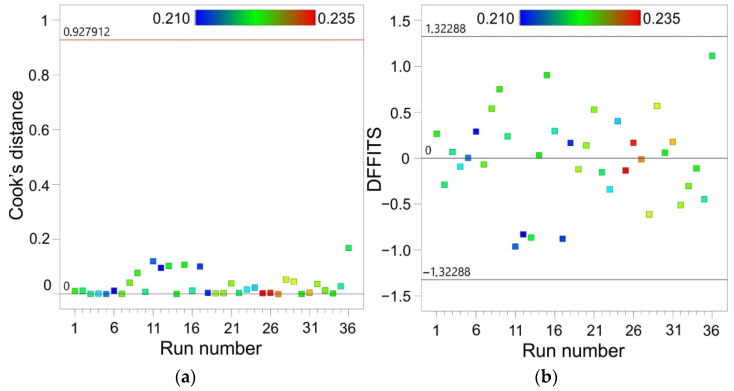
(**a**) Cook’s distances and (**b**) difference of fits vs. run number.

All distance values were of the same order and did not exceed the threshold value determined for the analysed data set (Figure 10a). All cases had no significant effect on the weighting of the regression equation.

Parameter DFFITS (difference of fits) measure the effect of the i-th observation on the predicted value using the regression equation ${\bar{y}}_{i}$, when this observation is omitted from the regression analysis. DFFITS can be explained by the following formula:

(9) $$\mathrm{DFFITS}=e_{i}\frac{h_{i}}{1-h_{i}}$$

All the points in the statistical regression were influential as they were located within the critical range (Figure 10b), so the model is adequate.

Due to complex interrelations between the input process parameters and the resultant COF, it is very hard to experimentally find the synergistic interactions of these parameters. Analysis of variance as a statistical method is an effective tool for understanding the qualitative and quantitative relationships between parameters.

**Figure 11:**
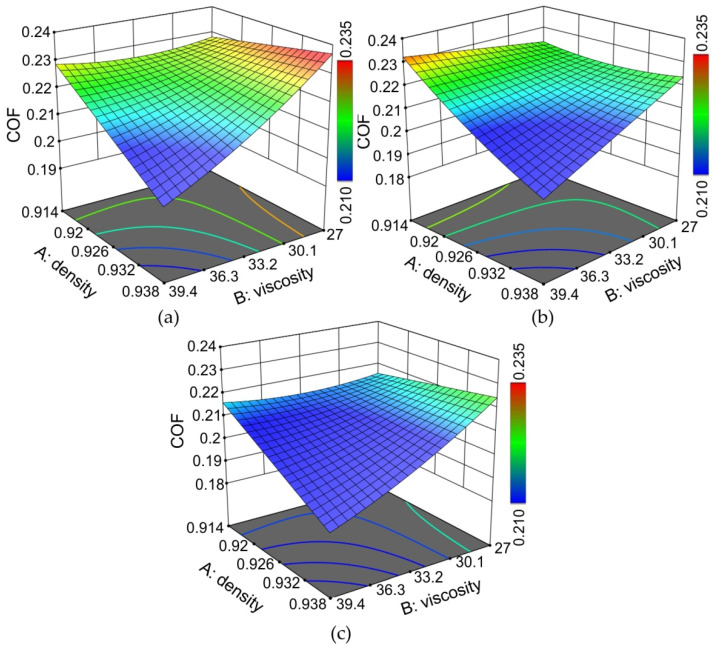
Response surface plots presenting the interaction between the density and viscosity of oils affecting the COF at (**a**) the minimum pressure considered (*p* = 2 MPa), (**b**) the average pressure considered (*p* = 7 MPa) and (**c**) the maximum pressure considered (*p* = 12 MPa).

The effect of the density and viscosity of the oil on the coefficient of friction was, in general, independent of the value of the pressure (Figure 11). In the whole range of pressures considered, the increase in the viscosity of the oil caused a reduction in the value of the COF. The effect of oil density on the COF value was similar. The most unfavourable friction conditions occurred when there was low density and low viscosity of the oils at the same time.

The lower the viscosity of the oil, the more fluid the oil, and the working layer of the oil (the so-called “oil film”) becomes thinner and thinner. In the case of hydrodynamic lubrication with a large number of closed lubricant pockets, the thickness of the oil film depends on the viscosity of the oil, the roughness of the contacting surfaces and the load. Increasing the viscosity of the oil under conditions of the elastic deformation of the surface asperities under load can lead to constituting the conditions of elastohydrodynamic lubrication [54].

Vegetable oils typically contain about 80–95% of fatty acids, which are one of the main performance improvers in lubricants [37]. The triglyceride structure of the oils tested provides qualities desirable in a lubricant. Triglycerides are glycerol molecules with three long chain fatty acids attached at the hydroxyl groups via ester linkages [55].

**Figure 12:**
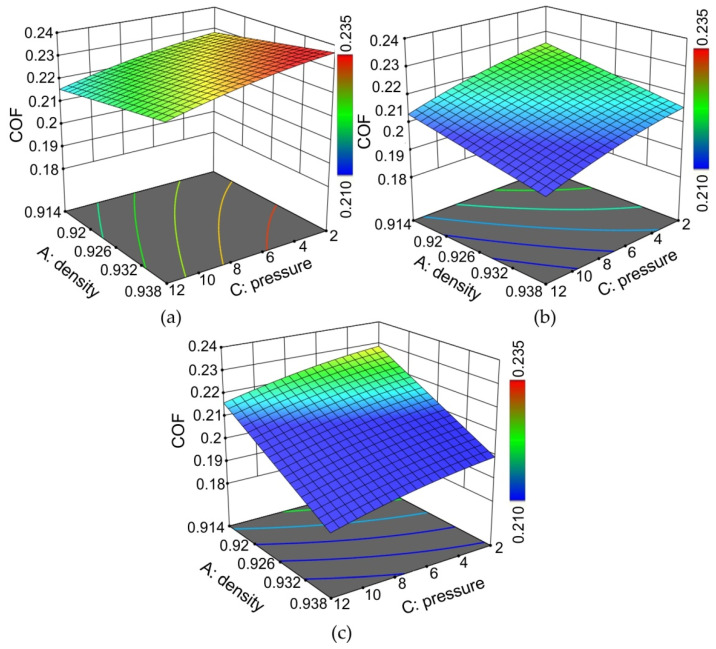
Response surface plots presenting the interaction between the density of the oils and the nominal pressures affecting the COF at (**a**) the minimum viscosity considered (η_k_ = 27 mm^2^/s), (**b**) the average viscosity considered (η_k_ = 32.15 mm^2^/s) and (**c**) the maximum viscosity considered (η_k_ = 39.4 mm^2^/s).

The effect of the nominal pressure on the value of the COF depended on the viscosity of the oil (Figure 12). In general, the increase in the nominal pressure led to a decrease in the COF in the range of the pressures considered (*p* = 2–12 MPa). This phenomenon has been observed by other authors [24,56,57] and is explained by the dependence of the friction on the clamping (normal) force in the SDT, where beyond a certain load, the relationship between the friction force and clamping force is nonlinear [57]. Moreover, it has been mentioned that in lubricated conditions, the increase in load pressure increases the pressure of the lubricant in closed oil pockets, allowing the metallic contacts of the surface asperities to be minimised. It should be highlighted that the frictional phenomena in SMF are different from the tribological phenomena, which exist in the kinematic couplings of machines (bearings, gears, etc.), where the hardness of both contacting surfaces is similar and the mechanism of the wearing-in phenomenon improves the cooperation of the two bodies. In SMF, one friction pair (tool) intentionally has much higher hardness that that of the sheet metal. Therefore, in such conditions, the contact area plays a key role in the frictional resistances resulting from the roughening and flattening mechanisms. The results of Emments [58], who analysed the effect of contact areas on the COF in the SDT, show that the contact area has a high influence on the COF. At the lowest considered value of oil viscosity (Figure 12a), the increase in the density of the oil caused a slight increase in the COF. However, the greater the viscosity of oil, the faster the COF value decreases with increasing density (Figure 12b,c). The most unfavourable conditions existed at low pressure and a low value of the COF.

**Figure 13:**
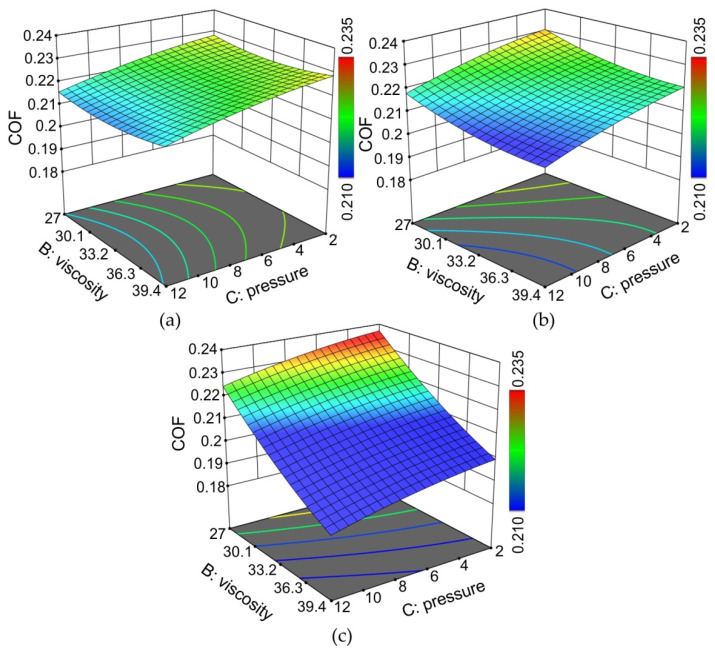
Response surface plots presenting the interaction between the viscosity of the oils and the nominal pressures affecting the COF at (**a**) the minimum density considered (ρ = 0.914 g/cm^3^), (**b**) the average density considered (ρ = 0.9205 g/cm^3^) and (**c**) the maximum density considered (ρ = 0.938 g/cm^3^).

Figure 13 presents the response surface plots presenting the interaction between the viscosity of the oils and the nominal pressure affecting the COF. At the lowest considered oil density (Figure 13a), the value of the COF was the most similar for the entire range of changes in both viscosity and pressure. As the density of the lubricant increased, the effect of oil viscosity on the COF was more pronounced. The highest values of the COF occurred for the lowest values of pressure and viscosity (Figure 13c). Viscosity, as the most important property of the oil, indicated resistance to flow, and was directly related to film formation and pressure [59]. Mobarak et al. [60] concluded that vegetable lubricants have better anti-wear properties than mineral oils. Compared with mineral lubricants, vegetable oils generally exhibit better lubricity and low evaporative losses [61,62]. Moreover, high temperatures ensure the maintenance of the thickness of the oil film, which guarantees the separation of the metallic contact of the mating surfaces [59]. Long fatty acid chains provide both boundary and hydrodynamic lubrication [58].

With regard to the above results of preliminary investigations, vegetable oil-based lubricants seem to be a good solution for use in cold metal forming. Palm, sunflower and cotton seed oils ensured the best efficiency of lubrication in terms of nominal pressures between 2 and 12 MPa. The obtained reduction in the value of the COF in general corresponded to the effectiveness of synthetic lubricants [24,57]. In contrast, linseed and coconut oils turned out to be the least effective in reducing the value of the COF. Further research is required to determine the effect of material deformation on the lubrication efficiency of vegetable oils. Sheet deformation as a result of the work hardening phenomenon causes a change in the topography and mechanical properties of the surface asperities. Thus, the conditions for constitution and volume change in the oil pockets due to deformation will be different. It would also be advisable to determine the effect of additives of hard nanopowders to vegetable oils on the value of the friction coefficient. Hard particles act like a third body, separating tool and workpiece surfaces. However, their beneficial effect depends on the type of oil in which they are suspended.

## 4. Summary

In this paper, six kinds of vegetable oils were used to study the effect of lubricant type on the value of the COF of DC04 steel sheet in the SDT. Experimental observations and the analysis of variance allowed the following quantitative and qualitative conclusions to be drawn:

- The following vegetable oils ensured the best efficiency of sheet metal lubrication in terms of nominal pressures between 2 and 12 MPa: palm, sunflower and cotton seed; these oils decreased the value of the COF by about 11–16% depending on the nominal pressure.
- Linseed and coconut oils had the most unfavourable lubrication properties, reducing the COF by about 7–12% depending on the nominal pressure.
- The small difference between the predicted R^2^ and the adjusted R^2^ (0.0187) and the F-value of 78.41 indicated that the polynomial multiple regression model is adequate.
- In the ANOVA model, the correlation of both density and viscosity was a significant factor. Load pressure was the most significant factor at a probability level *p* < 0.0001.
- In the whole range of pressures considered, the increase in the viscosity of the oil caused a reduction in the value of the COF. The effect of oil density on the COF value was similar.
- The most unfavourable friction conditions occurred when there was low density and low viscosity of the oils at the same time.
- At the lowest considered value of oil viscosity (η_k_ = 27 mm^2^/s), the increase in the density of the oil caused a slight increase in the COF. However, the greater the viscosity of the oil, the faster the COF value decreased with increasing density.

## Figures and Tables

**Table 1:** Factors included in analysis of variance.

Parameter	Density ρ, g/cm^3^	Kinematic Viscosity ηk, mm^2^/s	Nominal Load, MPa
Levels of variability	0.914, 0.916, 0.918, 0.919, 0.938	27, 27.5, 29, 34, 36, 39.4	2, 4, 6, 8, 10,12

**Table 2:** Results of the ANOVA for the polynomial regression model *.

Source	SS	DOF	Mean Square	F-Value	*p*-Value	Meaning
Model	0.0013	6	0.0002	78.41	<0.0001	significant
*A*—Density	7987 × 10^−6^	1	7987 × 10^−6^	2.86	0.1014	
*B*—Viscosity	0.0001	1	0.0001	19.87	0.0001	
*C*—Pressure	0.0007	1	0.0007	237.36	<0.0001	
AB	0.0000	1	0.0000	8.03	0.0083	
B^2^	0.0000	1	0.0000	6.90	0.0136	
C^2^	0.0000	1	0.0000	4.65	0.0394	
Residual	0.0001	29	2.79 × 10^−6^			
Correlation Total	0.0014	53				

* (SS—sum of squares; DOF—degree of freedom).

**Table 3:** Fit statistics of the regression model.

Standard Deviation	0.0017	R^2^	0.9419
Mean	0.2217	Adjusted R^2^	0.9299
Coefficient of variance. %	0.7536	Predicted R^2^	0.9112
		Adequacy precision	33.9435

## Data Availability

The data presented in this study are available on request from the corresponding author.
